# Mesothelin as a biomarker for targeted therapy

**DOI:** 10.1186/s40364-019-0169-8

**Published:** 2019-08-23

**Authors:** Jiang Lv, Peng Li

**Affiliations:** 1 Key Laboratory of Regenerative Biology, South China Institute for Stem Cell Biology and Regenerative Medicine, Guangzhou Institutes of Biomedicine and Health, Chinese Academy of Sciences, Guangzhou, China; 2 Guangdong Provincial Key Laboratory of Stem Cell and Regenerative Medicine, South China Institute for Stem Cell Biology and Regenerative Medicine, Guangzhou Institutes of Biomedicine and Health, Chinese Academy of Sciences, Guangzhou, China; 3 University of Chinese Academy of Sciences, Shijingshan District, Beijing, China

**Keywords:** Mesothelin, Biomarker, Targeted therapy, Immunotherapy, CAR-T

## Abstract

CAR-T cell therapy targeting CD19 has achieved remarkable success in the treatment of B cell malignancies, while various solid malignancies are still refractory for lack of suitable target. In recent years, a large number of studies have sought to find suitable targets with low “on target, off tumor” concern for the treatment of solid tumors. Mesothelin (MSLN), a tumor-associated antigen broadly overexpressed on various malignant tumor cells, while its expression is generally limited to normal mesothelial cells, is an attractive candidate for targeted therapy. Strategies targeting MSLN, including antibody-based drugs, vaccines and CAR-T therapies, have been assessed in a large number of preclinical investigations and clinical trials. In particular, the development of CAR-T therapy has shown great promise as a treatment for various types of cancers. The safety, efficacy, doses, and pharmacokinetics of relevant strategies have been evaluated in many clinical trials. This review is intended to provide a brief overview of the characteristics of mesothelin and the development of strategies targeting MSLN for solid tumors. Further, we discussed the challenges and proposed potential strategies to improve the efficacy of MSLN targeted immunotherapy.

## Background

### The discovery and function of MSLN

The MSLN gene encodes a 71-KD precursor, which is a glycosylphosphatidylinositol (GPI)-anchored membrane glycoprotein that is cleaved into two products at arginine 295 (Arg295): a soluble 31-KD N-terminal protein called megakaryocyte potentiating factor (MPF) and a 40-KD membrane-bound fragment called MSLN (mesothelin). Both MPF and MSLN are bioactive, but their exact functions remain unclear. MPF was initially reported to stimulate megakaryocyte colony formation in the presence of interleukin-3 in mice but not alone [1], while its activity is unknown in humans. MSLN was first described as a membrane protein expressed on mesothelioma and ovarian cancer cells [2] and normal mesothelial cells [2, 3]. A previous study showed that MSLN seemed to be a nonessential component in normal cells, as MSLN knockout mice did not present with abnormal development or reproduction [4]. In contrast, preclinical and clinical studies showed that aberrant MSLN expression on tumor cells plays an important role in promoting proliferation and invasion [5]. MSLN has also been identified as a receptor of CA125 that mediates cell adhesion [6]. The interaction of CA125 and MSLN play an important role in ovarian cancer cell peritoneal implantation and increase the motility and invasion of pancreatic carcinoma cells [7–9]. The overexpression of MSLN could activate the NFκB, MAPK, and PI3K pathways and subsequently induce resistance to apoptosis [10] or promote cell proliferation, migration, and metastasis by inducing the activation and expression of MMP7 [9] and MMP9 [5]. An increase in tumor burden and poor overall survival are associated with elevated MSLN expression according to clinical observations [11, 12]. Structural prediction revealed that a superhelical structure with armadillo-type repeats constitutes a part of its three-dimensional structure [13], and the structure of an N-terminal fragment that binds to the Fab SS1 antibody has been clarified [14], but the structure of the whole protein is still unclear.

### Expression of MSLN in malignant cells and prognosis

Generally, MSLN is expressed on normal mesothelial cells in the pleura, pericardium, and peritoneum and in epithelial cells on the surface of the ovary, tunica vaginalis, rete testis, and fallopian tubes in trace amounts [3]. In contrast, the aberrant overexpression of MSLN is observed in various cancer cells. MSLN was initially characterized in mesothelioma and ovarian cancer by Chang et al. with the mAb K1 [15]. Chang and colleagues found that MSLN was present in 10 of 15 nonmucinous ovarian cancers and absent in all 4 mucinous ovarian cancers examined [2]. In addition, all 15 cases of epithelial mesothelioma, but none of the 4 cases of sarcomatous mesothelioma, expressed MSLN [16]. This was in line with the results of another independent study that confirmed MSLN reactivity in all 44 epithelioid mesotheliomas and in the epithelial components of 3 biphasic mesotheliomas, but not in any of 8 sarcomatous mesotheliomas examined [17]. According to the statistics in this study, MSLN was present in 15 of 48 (31%) lung cancers (adenocarcinomas (12/31) and squamous carcinomas (limited, 3/17)) and in 42 of 86 (49%) nonpulmonary adenocarcinomas (ovary (14/14), peritoneum (5/5), endometrium (6/9), pancreas (10/11), stomach (2/4), and colon (5/16); none of 12 breast, 9 kidney, 4 thyroid, and 2 prostate cancers showed evidence of MSLN) according to assays with the 5B2 anti-MSLN monoclonal antibody. MSLN was immunohistochemically evaluated in 596 lung carcinomas of different types by Miettinen M and Sarlomo-Rikala M in 2003 [18]. MSLN reactivity was observed in 78 of 148 (53%) adenocarcinomas, 29 of 124 (23%) squamous cell carcinomas and 15 of 118 (13%) large cell carcinomas but was absent in small cell carcinomas. These results suggest that MSLN could act as an immunohistochemical biomarker for the determination of the subtype classification of mesotheliomas and lung cancer to a certain degree because of its specific expression pattern in these two cancers. MSLN is expressed in the majority of pancreatic cancers, and independent studies revealed that almost 100% of pancreatic cancers are positive for MSLN but that normal pancreatic tissues did not show evidence of MSLN [3, 19, 20]. Subsequent studies demonstrated the expression of MSLN in a broad spectrum of solid tumors with distinct frequency and distribution patterns, including extrahepatic biliary cancers (95%), triple negative breast cancer (66%), endometrial carcinomas (59%), colorectal carcinomas (30%), cervical carcinomas (25%) and esophageal (46%), endometrial (89%) and thymic cancer [3, 21–26]. A recent study reported that 25.6% of 117 patients with gastric carcinoma showed high levels of MSLN expression, which was associated with a poor prognosis [27]. We also detected MSLN expression to different degrees in 9 gastric cancer tissues but not in normal gastric tissue [28]. The elevated expression of MSLN was correlated with poorer prognoses in patients with ovarian cancer [29], cholangiocarcinoma [30, 31], lung adenocarcinoma [29, 32], triple negative breast cancer [4, 33] and resectable pancreatic adenocarcinoma [34–36].

In addition, MSLN is shed into the serum of patients with solid tumors, in which it is referred to as soluble MSLN-related protein (SMRP) [37]. The production of SMRP could be associated with abnormal splicing, which results in a secreted form or its cleavage from the membrane by the TNFα-converting enzyme ADAM17 [38]. SMRP was also identified as a promising cancer biomarker in the sera of patients with mesothelioma, in which elevated SMR levels in serum was correlated with advanced stage and increased disease burden [37, 39]. However, the sensitivity and specificity of SMRP as a tumor marker in ovarian cancer was limited [40]. The value of soluble MSLN in diagnosis and the prediction of cancer progression remains to be determined, and its combination with other tumor markers may be more precise for diagnosis.

## Targeted therapy

**Fig. 1:**
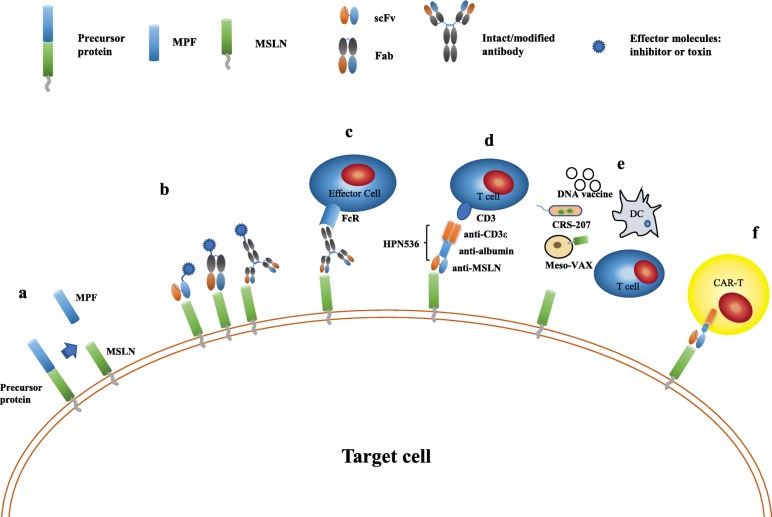
MSLN-targeted therapy strategies. **a**, the precursor protein is cleaved into two products, i.e. soluble protein MPF and GPI-anchored membrane protein MSLN; **b**, anti-MSLN antibody derived scFv, Fab, or intact/modified antibody are conjugated with the effector molecules (inhibitor or toxin) and induce cell death after binding to tumor cells; **c**, the binding of amatuximab to MSLN expressed on tumor cell membrane leads to ADCC; **d**, HPN536 directs T cells to kill tumor cells expressing MSLN; **e**, cancer vaccines arouse tumor specific immune response; **f**, the T cells are engineering to express CAR and redirected to tumor cells

Given that MSLN expression is rather limited in several normal tissues but highly elevated in the solid tumors mentioned above, MSLN is a potential target for antigen-specific therapy (Fig. 1).

### Antibody-based drugs

Antibody-based drugs are used to target and kill tumor cells via neutralization by antibodies, antibody-dependent cell-mediated cytotoxicity (ADCC), antibody-dependent cell-mediated phagocytosis (ADCP) or antibodies conjugated with effector molecules (toxins or inhibitors), which mediate apoptosis or suppress cell proliferation.

The specific uptake of the indium^111−^labeled MSLN antibody K1 by tumor cells was observed by Hassen et al. [41]. The conjugation of a fragment of *Pseudomonas* exotoxin A (PE) to this antibody resulted in cytotoxicity in MSLN-expressing cell lines and tumor regression in tumor-bearing mice [42]. A new murine-derived antibody with higher affinity termed SS1 was produced via phage display and hotspot mutagenesis [43, 44]. The fusion of the PE38 portion to SS1 resulted in a recombinant immunotoxin (RIT) termed SS1P, which enters cells by receptor-mediated endocytosis and induces apoptosis by inactivating elongation factor 2 to impede protein synthesis [45]. Many drugs based on the MSLN antibody SS1 or other modified and humanized versions have been developed for targeted therapy (Table 1).

**Table 1:** Clinical trials for MSLN-targeted therapies based on antibody-based drugs and vaccines

Agent	NCT Number (Reference)	Title	Status (Results)	Interventions	Phases	Enrollment	Start Date	Locations
SS1P	NCT01445392 [46]	SS1(dsFV)PE38 Plus Pemetrexed and Cisplatin to Treat Malignant Pleural Mesothelioma	Terminated	Biological: Multicycle SS1P Drug: Pemetrexed Drug: Cisplatin Biological: Single cycle SS1PBiological: Multicycle SS1P Drug: Pemetrexed Drug: Cisplatin Biological: Single cycle SS1PBiological: Multicycle SS1P Drug: Pemetrexed Drug: Cisplatin Biological: Single cycle SS1P	Phase 1	24	2007-11-14	United States
	NCT01362790 [47]	SS1P and Pentostatin Plus Cyclophosphamide for Mesothelioma	Unknown status (Results Submitted)	Drug: Pentostatin; Drug: Cyclophosphamide; Biological: SS1(dsFv)PE38 - lot 073I0809; Biological: SS1(dsFv)PE38 - lot FIL129J01	Phase 1/2	55	2011-05-11	United States
	NCT01051934	A Phase I Trial of SS1 (dsFv) PE38 With Paclitaxel, Carboplatin, and Bevacizumab in Subjects With Unresectable Non-Small Cell Lung Adenocarcinoma	Completed	Drug: SS1 (dsFv) PE38; Drug: Paclitaxel; Drug: Carboplatin; Drug: Bevacizumab	Phase 1	2	2009-12-29	United States
	NCT00066651 [48]	Immunotoxin Therapy in Treating Patients With Advanced Solid Tumors	Completed	Biological: SS1(dsFv)-PE38 immunotoxin	Phase 1		2003-07-01	United States
	NCT00006981 [49]	Immunotoxin Therapy in Treating Patients With Advanced Cancer	Completed	Biological: SS1(dsFv)-PE38 immunotoxin	Phase 1		2000-12-01	United States
Amatuximab	NCT02357147 [50]	Study of the Safety and Efficacy of Amatuximab in Combination With Pemetrexed and Cisplatin in Subjects With Unresectable Malignant Pleural Mesothelioma (MPM)	Terminated	Drug: Placebo; Drug: Amatuximab; Drug: Pemetrexed; Drug: Cisplatin	Phase 2	108	2015-11-03	Australia; France; Germany; Italy; United Kingdom; United States
	NCT01521325	A Single-Dose Pilot Study of Radiolabeled Amatuximab (MORAb-009) in Mesothelin Over Expressing Cancers	Completed	Drug: Amatuximab	Phase 1	6	2011-09-01	United States
	NCT01413451	Amatuximab for High Mesothelin Cancers	Terminated	Drug: Amatuximab (MORab-009)	Early Phase 1	7	2011-07-12	United States
	NCT01018784 [51]	A Study of MORAb-009 in Patients With Solid Tumor	Completed	Drug: MORAb-009	Phase 1	17	2009-11-01	Japan
	NCT00738582 [52–54]	An Efficacy Study of MORAb-009 (Amatuximab) in Subjects With Pleural Mesothelioma	Completed (Results Submitted)	Drug: MORAb-009(Amatuximab); Drug: Pemetrexed; Drug: Cisplatin	Phase 2	89	2008-12-01	Canada; Germany; Netherlands; Spain; United States
	NCT00570713 [52]	An Efficacy Study of MORAb-009 in Subjects With Pancreatic Cancer	Completed (Results Available)	Drug: MORAb-009; Drug: Placebo; Drug: Placebo; Drug: Gemcitabine	Phase 2	155	2007-12-01	Belgium; Canada; Germany; Spain; United States
	NCT00325494	A Study of MORAb-009 in Subjects With Pancreatic Cancer, Mesothelioma, or Certain Types of Ovarian or Lung Cancer	Completed	Drug: MORAb-009	Phase 1	24	2006-05-01	United States
Anetumab ravtansine (BAY94–9343)	NCT03816358	Anetumab Ravtansine With Nivolumab, Ipilimumab and Gemcitabine Hydrochloride in Treating Patients With Mesothelin Positive Advanced Pancreatic Cancer	Suspended	Biological: Anetumab Ravtansine; Drug: Gemcitabine Hydrochloride; Biological: Ipilimumab; Biological: Nivolumab	Phase 1	64	2019-07-01	Canada
	NCT03455556	Anetumab Ravtansine and Atezolizumab in Treating Participants With Advanced Non-small Cell Lung Cancer	Recruiting	Biological: Anetumab Ravtansine; Biological: Atezolizumab; Other: Laboratory Biomarker Analysis	Phase 1/2	49	2018-08-10	United States
	NCT03126630	Pembrolizumab With or Without Anetumab Ravtansine in Treating Patients With Mesothelin-Positive Pleural Mesothelioma	Recruiting	Biological: Anetumab Ravtansine; Other: Laboratory Biomarker Analysis; Biological: Pembrolizumab; Other: Pharmacological Study	Phase 1/2	134	2018-02-08	United States; Canada
	NCT03102320	Phase 1b Multi-indication Study of Anetumab Ravtansine in Mesothelin Expressing Advanced Solid Tumors	Recruiting	Drug: Cisplatin; Drug: Gemcitabine; Drug: Anetumab ravtansine (BAY94–9343)	Phase 1	348	2017-05-26	United States; Australia; Belgium; Canada; France; Germany; Italy; Korea; Netherlands; Singapore; Spain; Switzerland; United Kingdom
	NCT03023722	Phase II Anetumab Ravtansine in Pre-treated Mesothelin-expressing Pancreatic Cancer	Recruiting	Drug: anetumab ravtansine	Phase 2	30	2017-05-11	United States
	NCT02824042	Thorough ECG (Electrocardiogram) and Drug Interaction Study With Anetumab Ravtansine and Itraconazole	Active, not recruiting	Drug: Anetumab ravtansine (BAY94–9343); Drug: Itraconazole	Phase 1	63	2016-09-12	United States; Australia; Belgium; France; Netherlands; Spain
	NCT02839681	Anti-Mesothelin Antibody Drug Conjugate Anetumab Ravtansine for Mesothelin Expressing Lung Adenocarcinoma	Terminated (Results Submitted)	Drug: Anetumab Ravtansine; Device: Blood test	Phase 2	2	2016-07-19	United States
	NCT02751918 [55]	Phase Ib Study of Anetumab Ravtansine in Combination With Pegylated Liposomal Doxorubicin in Patients With Recurrent Mesothelin-expressing Platinum-resistant Cancer	Recruiting	Drug: Anetumab ravtansine (BAY94–9343); Drug: Pegylated Liposomal Doxorubicin	Phase 1	71	2016-06-08	United States; Belgium; France; Japan; Moldova; Spain
	NCT02696642	Phase I Study of Anetumab Ravtansine in Hepatic or Renal Impairment	Active, not recruiting	Drug: Anetumab ravtansine (BAY94–9343)	Phase 1	54	2016-04-14	France; Moldova
	NCT02639091	Phase Ib Study of Anetumab Ravtansine in Combination With Pemetrexed and Cisplatin in Mesothelin-expressing Solid Tumors	Active, not recruiting	Drug: BAY 94–9343; Drug: Pemetrexed; Drug: Cisplatin	Phase 1	36	2016-02-03	United States; Italy
	NCT02610140	Phase II Anetumab Ravtansine as 2nd Line Treatment for Malignant Pleural Mesothelioma (MPM)	Active, not recruiting	Drug: Anetumab ravtansine (BAY 94–9343); Drug: Vinorelbine	Phase 2	248	2015-12-03	United States; Australia; Belgium; Canada; Finland; France; Italy; Korea; Netherlands; Poland; Russian Federation; Spain; Turkey; United Kingdom
	NCT02485119	Phase I Dose Escalation Study of BAY94–9343 Given by Intravenous Infusion Every 3 Weeks in Japanese Subjects With Advanced Malignancies	Completed	Drug: BAY94–9343	Phase 1	12	2015-08-14	Japan
	NCT01439152 [56]	Phase I Study to Determine the Maximum Tolerable Dose of BAY94–9343 in Patients With Advanced Solid Tumors.	Active, not recruiting	Drug: BAY94–9343; Drug: BAY94–9343 (Expansion); Drug: BAY94–9343 (1.8 mg/kg); Drug: BAY94–9343 (2.2 mg/kg)	Phase 1	147	2011-09-07	United States
DMOT4039A	NCT01469793	A Study of DMOT4039A in Participants With Unresectable Pancreatic or Platinum-Resistant Ovarian Cancer	Completed	Drug: DMOT4039A	Phase 1	71	2011-11-01	United States
	NCT01832116	89Zr-MMOT PET Imaging in Pancreatic and Ovarian Cancer Patients	Completed	Drug: 89Zr-MMOT0530A	Phase 1	11	2013-03-01	Netherlands
BMS-986148	NCT02884726 [57]	Phase 1 Study of Mesothelin-ADC	Completed	Drug: BMS-986148	Phase 1	8	2016-10-14	Japan
	NCT02341625 [57]	A Study of BMS-986148 in Patients With Select Advanced Solid Tumors	Active, not recruiting	Drug: BMS-986148; Biological: Nivolumab	Phase 1/2	407	2015-06-17	United States; Australia; Belgium; Canada; Italy; Netherlands; United Kingdom
LMB-100	NCT03644550	Anti-Mesothelin Immunotoxin LMB-100 Followed by Pembrolizumab in Malignant Mesothelioma	Recruiting	Drug: LMB-100; Biological: Pembrolizumab	Phase 2	38	2018-12-04	United States
	NCT03436732	Mesothelin-Targeted Immunotoxin LMB-100 in Combination With SEL-110 in Subjects With Malignant Pleural or Peritoneal Mesothelioma	Suspended	Drug: LMB-100; Drug: SEL-110	Phase 1	23	2018-02-28	United States
	NCT02810418	Mesothelin-Targeted Immunotoxin LMB-100 Alone or in Combination With Nab-Paclitaxel in People With Previously Treated Metastatic and/or Locally Advanced Pancreatic Ductal Adenocarcinoma and Mesothelin Expressing Solid Tumors	Active, not recruiting	Drug: LMB-100; Drug: Nab-Paclitaxel; Device: Mesothelin Expression	Phase 1/2	40	2016-08-03	United States
	NCT02798536	Mesothelin-Targeted Immunotoxin LMB-100 in People With Malignant Mesothelioma	Active, not recruiting	Drug: LMB-100; Drug: nab-paclitaxel	Phase 1	21	2016-06-10	United States
BAY2287411	NCT03507452	First-in-human Study of BAY2287411 Injection, a Thorium-227 Labeled Antibody-chelator Conjugate, in Patients With Tumors Known to Express Mesothelin	Recruiting	Drug: BAY2287411	Phase 1	228	2018-06-13	United States; Finland; Netherlands; Sweden; United Kingdom
HPN536	NCT03872206	Study of HPN536 in Patients With Advanced Cancers Associated With Mesothelin Expression	Recruiting	Biological: HPN536	Phase 1/2	87	2019-04-16	United States
CRS-207	NCT02004262	Safety and Efficacy of Combination Listeria/GVAX Pancreas Vaccine in the Pancreatic Cancer Setting	Completed (Results Available)	Biological: GVAX Pancreas Vaccine; Biological: CRS-207; Drug: Chemotherapy; Drug: cyclophosphamide	Phase 2	303	2014-02-05	Canada
	NCT01417000 [58]	Safety and Efficacy of Combination Listeria/GVAX Immunotherapy in Pancreatic Cancer	Completed (Results Available)	Biological: GVAX Pancreas; Biological: CRS-207; Drug: Cyclophosphamide	Phase 2	93	2011-09-21	United States
	NCT00585845 [59]	Study of Safety and Tolerability of Intravenous CRS-207 in Adults With Selected Advanced Solid Tumors Who Have Failed or Who Are Not Candidates for Standard Treatment	Terminated	Biological: CRS-207, Live-attenuated *Listeria monocytogenes* expressing human Mesothelin	Phase 1	17	2007-12-01	United States; Israel
JNJ-64041757	NCT03371381	An Efficacy and Safety Study of JNJ-64041757, a Live Attenuated Listeria Monocytogenes Immunotherapy, in Combination With Nivolumab Versus Nivolumab Monotherapy in Participants With Advanced Adenocarcinoma of the Lung	Terminated	Biological: JNJ-64041757; Drug: Nivolumab	Phase 1/2	12	2018-01-02	United States; Belgium; Spain
	NCT02592967	Safety & Immunogenicity of JNJ-64041757, Live-attenuated Double-deleted Listeria Immunotherapy, in Subjects With Non Small Cell Lung Cancer	Terminated	Biological: JNJ-64041757(Cohort 1A and 1B);Biological: JNJ-64041757(Cohort 2A and 2B)	Phase 1	18	2015-12-02	United States
Neoantigen DNA Vaccine	NCT03122106	Neoantigen DNA Vaccine in Pancreatic Cancer Patients Following Surgical Resection and Adjuvant Chemotherapy	Recruiting	Biological: Personalized neoantigen DNA vaccine; Device: TDS-IM Electrode Array System; Procedure: Peripheral blood draws	Phase 1	15	2018-01-05	United States

### SS1P

SS1P has been tested in several clinical trials that enrolled patients with advanced cancers. In an early phase I clinical trial (NCT00066651) [48], the dose-limiting toxicities (DLTs), maximum tolerated dose (MTD) and pharmacokinetics (PK) of SS1P were tested in 34 patients with mesothelioma (*n* = 20), ovarian cancer (*n* = 12) and pancreatic cancer (*n* = 2). With a limited sample size, this clinical trial demonstrated that the combination of SS1P with prednisone can reduce the risk of toxicity due to SS1P and allow the use of an increased drug dosage. No significant pericardial toxicity was observed in any of the patients, which suggested that the MSLN antibody SS1P presented less risk to pericardial mesothelial cells. Among the 33 evaluable patients, 4 had a partial response (PR), 19 had stable disease (SD), and 10 had progressive disease (PD). However, SS1P was proven to be immunogenic in a later clinical trial (NCT00006981) [49]. Twenty-four patients with chemo-resistant solid tumors received SS1P treatment at dosages of 4, 8, 12, 18, and 25 μg/kg/day (× 10). One patient had a PR, 12 had SD, and 11 had PD. It is noteworthy that high levels of neutralizing antibodies against SS1P were detected in 75% of patients, which could undermine the anti-tumor efficacy.

Given that the administration of SS1P alone showed a moderate effect, the combination therapy might be more effective. In the clinical trial NCT01362790 [47], 10 patients with chemotherapy-refractory mesothelioma received SS1P in combination with pentostatin and cyclophosphamide. Three patients had a PR (44% ~ 74%), 3 had SD and 4 had PD. Adverse events were evaluated for all patients. Grade 3 toxicities, including noncardiac chest pain, pleuritic pain, and back pain (9% each) were observed, but no grade 4 toxicities were observed in patients. Meanwhile, adverse events associated with pentostatin or cyclophosphamide, such as grade 4 lymphopenias, were observed in all patients. In contrast to the trials described above, the involvement of pentostatin and cyclophosphamide delayed the formation of neutralizing antibodies to SS1P, thereby allowing a prolonged period of therapy. SS1P combined with pemetrexed and cisplatin was further tested for treating chemotherapy-naive patients with advanced malignant pleural mesothelioma (MPM) (NCT01445392) [46]. Of the 20 evaluable subjects, 12 patients had a PR, 3 had SD, and 5 had PD. Notably, the changes in the relative serum levels of MSLN, MPF and CA125 were significantly correlated with responses (PR > SD > PD). These biomarker levels were generally decreased in 12 patients who received PR but were substantially increased in 5 patients who had PD.

### Amatuximab

Amatuximab (MORAb-009) is a chimeric monoclonal antibody consisting of the SS1 scFv fused to the human IgG1 and κ constant regions. The binding of amatuximab to MSLN expressed on tumor cell surfaces leads to ADCC.

Two clinical trials (NCT00570713 and NCT00738582) showed that no severe (grade 3 or 4) drug hypersensitivity adverse events (DHAEs) were observed in any of the subjects. Among the 20 of 24 patients evaluable for response, none had complete or partial responses, and only 11 patients had SD and 9 had PD [52]. MORAb-009 treatment resulted in a remarkable elevation in serum CA125 levels in all 8 patients under surveillance, possibly due to the interruption of binding between MSLN and CA125 by amatuximab, which could prevent the intraperitoneal/intrapleural metastasis of ovarian cancer and mesothelioma [53]. A clinical trial (NCT01018784) in Japanese patients with mesothelioma, pancreatic adenocarcinoma or other MSLN-positive solid tumors revealed that the weekly single administration of amatuximab in 4-week cycles at increasing doses ranging from 50 to 200 mg/m^2^ led to limited treatment effects. Three of the 17 patients had SD, and 14 had PD [51].

The anti-tumor effect of amatuximab in combination with pemetrexed and cisplatin was elevated in 89 patients at 26 centers (NCT00738582) [54]. Amatuximab in combination with pemetrexed and cisplatin was administered according to the response (PR or SD) for up to 6 cycles. Thirty-three patients had a PR, and 42 had SD. The detection of the change in the MPF level in serum before and after treatment in 59 patients also showed that the decreased MPF level was correlated with good prognosis. The combination therapy led to severe adverse events, including hypersensitivity reactions, neutropenia, and atrial fibrillation. Dyspnea and fatigue were observed during the maintenance phase.

An ^111^Indium (^111^In) radiolabel was used to characterize the biodistribution and dosimetry of amatuximab in 6 patients (4 with malignant mesothelioma and 2 with pancreatic adenocarcinoma) [60]. SPECT/CT imaging showed ^111^In-amatuximab uptake in both primary tumors and metastatic sites and that uptake was increased in mesothelioma compared with that in pancreatic cancer. Notably, ^111^In-amatuximab uptake in the heart, liver, kidneys and spleen was also confirmed. Even so, amatuximab was generally well tolerated. Amatuximab PK was characterized in the clinical trial NCT02357147. It revealed that higher amatuximab exposure in combination with chemotherapy was associated with prolonged OS [50].

### Anetumab ravtansine

Anetumab ravtansine, also referred to as BAY94–9343, is a human anti-MSLN antibody fused to DM4, which is a maytansinoid tubulin inhibitor that mainly affects proliferating cells. The specific binding of BAY94–9343 to MSLN with high affinity induces efficient antigen internalization. BAY94–9343 showed dose-dependent anti-tumor efficacy and bystander effects in xenogeneic tumor models [56]. The antitumor efficacy of anetumab ravtansine in combination with pegylated liposomal doxorubicin (PLD), carboplatin, copanlisib and bevacizumab was investigated for the treatment of ovarian cancer. The involvement of combination therapy showed enhanced anti-proliferative activity and increased apoptosis in vitro and improved in vivo efficacy in tumor-bearing mice [55]. The safety, tolerability, pharmacokinetics, and pharmacodynamics were then evaluated in clinical trials. Several phase 1/2 studies were carried out to explore the dosage and side effects of anetumab ravtansine when administered together with pemetrexed, cisplatin, PLD, itraconazole, gemcitabine, pembrolizumab, atezolizumab, gemcitabine hydrochloride, ipilimumab or nivolumab (Table 1). However, only one clinical trial data for anetumab ravtansine was submitted to ClinicalTrials.gov prior to the submission of this review.

### DMOT4039A

DMOT4039A is a humanized anti-MSLN mAb (h7D9.v3) fused to the antimitotic agent monomethyl auristatin E (MMAE) [61]. It inhibited cell proliferation at an IC50 of 0.3 nmol/L and regressed tumor growth in a dose-dependent manner in a mouse model. In another clinical trial (NCT01469793), DMOT4039A was administered to 71 patients with pancreatic cancer (*n* = 40) or ovarian cancer (*n* = 31) [62]. Fifty-four patients received a DMOT4039A injection every 3 weeks (2.4–2.8 mg/kg; q3w), and 17 patients received an injection weekly (0.8–1.2 mg/ kg). Hyperglycemia (grade 3) and hypophosphatemia (grade 3) were observed in 2 patients treated with DMOT4039A every 3 weeks at a dosage of 2.8 mg/kg but no DLTs were observed in patients treated with other dosages. Related severe adverse events occurred in 5 patients at a dosage of 2.4–2.8 mg/kg every 3 weeks and one patient at a dosage of 1.2 mg/kg weekly. Cumulative peripheral neuropathy (grades 1–3) was observed in 14 patients due to microtubule inhibitors. Six patients (4 ovarian cancer; 2 pancreatic cancer) treated with DMOT4039A at 2.4 to 2.8 mg/kg had a PR.

### BMS-986148

BMS-986148 is an antibody-drug conjugate that might be related to MDX-1204, which contains a MAb conjugated to the potent alkylating agent duocarmycin (MED2460) and causes cell death after internalization by target cells [57]. A clinical trial was carried out to evaluate the safety, tolerability, pharmacokinetics, immunogenicity, antitumor activity and pharmacodynamics of BMS-986148 administered alone and in combination with nivolumab in selected patients with mesothelioma, nonsmall cell lung cancer (NSCLC), ovarian cancer, pancreatic cancer and gastric cancer. This study aimed to enroll over 400 patients from 12 countries (NCT02341625). Another phase 1 clinical trial (NCT02884726) in Japan has been completed.

### LMB-100/ RG7787

LMB-100/ RG7787 is a re-engineered version of a humanized anti-MSLN Fab based on SS1 that is fused to a truncated and deimmunized PE24 moiety with higher activity and less immunogenicity [63]. LMB-100 inhibits protein synthesis [64] and is regulated by the tyrosine kinase DDR1 [65]. The addition of a DDR1 inhibitor resulted in the increased shrinkage of tumor xenografts. The antitumor efficacy of LMB-100 for pancreatic cancer, triple negative breast cancer (TNBC), and gastric cancer has been proven in preclinical studies [63, 66]. Its combination with actinomycin D [67], Nab-Paclitaxel [68], taxanes [69], and panobinostat [70] enhances its antitumor activity. LMB-100 is currently undergoing clinical testing in combination therapy in patients with MSLN-positive malignancies.

### BAY2287411

BAY2287411, a thorium-227-labeled antibody-chelator conjugate, was administered to patients with tumors known to express MSLN to evaluate the safety, tolerability, maximum tolerated dose, PK, anti-tumor activity and recommended dose for further clinical development (NCT03507452). This phase 1 study started in June 2018. More than 200 participants may eventually be enrolled with nonrandomized allocation. A recent study demonstrated that the combination of BAY2287411 with the damage response inhibitors ATRi and PARPi resulted in synergistic activity and increased anti-tumor efficacy [71].

### HPN536

HPN536 is the most recent MSLN-targeting antibody-based drug that is currently in clinical trials. It is a MSLN-targeting TriTAC and includes three domains: 1. an anti-MSLN domain that binds to MSLN-positive cells; 2. an anti-albumin domain antibody that extends its half-life; 3. an anti-CD3ε scFv that engages T cells [72]. HPN536 activates T cells in the presence of MSLN and directs T cells to kill cells expressing MSLN. It has a half-life of approximately 5 days and is well tolerated in cynomolgus monkeys subjected to a single treatment at a 10 mg/kg dosage. NCI-H292 tumor growth was impeded in mice implanted with human PBMCs and treated with HPN536. The associated phase 1/2a trial (NCT03872206) is a multicenter, open-label study designed to evaluate the safety, tolerability, PK and activity of HPN536 in up to 80 patients with advanced cancers associated with MSLN expression.

The short half-life and the immunogenicity of murine-derived antibodies and bacterial toxins have limited the efficacy of antibody-based drugs. To address these issues, novel humanized or fully human anti-MSLN antibodies and toxins with reduced immunogenicity need to be developed. Many studies have attempted to do this. The insertion of a disulfide bond to protect the furin cleavage site of SS1-PE24 improves its serum half-life and decreases its toxicity [73]. A study suggested that the involvement of albumin-binding domains could prolong the half-life and increase antitumor activity [74]. In addition, the removal of B- and T-cell epitopes from RIT led to greatly reduced antigenicity [75, 76]. Fully human antibodies were also developed and verified in preclinical studies [77, 78].

### Vaccines

Cancer vaccines are designed to induce tumor-specific immune responses in the host. A large number of studies have tested multiple platforms, including peptides, proteins, antigen presenting cells, tumor cells, and viral vectors [79]. The bacterium-based vaccine CRS-207, which uses a live-attenuated *Listeria monocytogenes* (*Lm)* strain ANZ-100 (*Lm* Δ*actA*/Δ*inlB*) engineered to express human MSLN, has been used to treat MSLN-positive cancers in clinical trials [59]. CRS-207 was evaluated in 17 subjects (7 with pancreatic ductal carcinoma (PDA), 5 with mesothelioma, 3 with NSCLC, and 2 with ovarian cancer) in a dose-escalation study with up to 4 doses (NCT00585845). CRS-207 was well tolerated at the top dose (1 × 10^9^ cfu). Immune activation was confirmed by a multiplexed serum cytokine assay and phenotype analysis. Thirty-seven percent of subjects survived ≥15 months, but none of them had a PR. CRS-207 has also been used in combination with low-dose cyclophosphamide and another vaccine, GVAX pancreas, which is derived from an irradiated allogeneic GM-CSF secreting cell line, in patients with metastatic PDA (NCT01417000) [58]. Sixty-one patients who received CRS-207 and Cy/GVAX had longer overall survival (6.1 months) than 29 patients treated with Cy/GVAX alone (3.9 months). A follow-up study to test the immune responses and efficacy produced by the combination of CRS-207 and the GVAX pancreas vaccine (with cyclophosphamide) compared to those produced by chemotherapy or CRS-207 alone in adults with previously treated metastatic pancreatic adenocarcinoma was conducted. The overall survival was 3.8 months for the cohort treated with Cy/GVAX + CRS-207, 5.4 months for the cohort treated with CRS-207 alone, and 4.6 months for the cohort treated with chemotherapy (NCT02004262).

JNJ-64041757 (previously referred to as ADU-214) is a live-attenuated, double-deleted (LADD) *Listeria monocytogenes* strain used as a potential treatment for NSCLC that was engineered by Aduro Biotech, Inc. in 2014. However, two clinical trials that attempted to evaluate its efficacy alone or in combination with nivolumab were both terminated due to a lack of clinical benefit (NCT02592967 and NCT03371381). A neoantigen DNA vaccine strategy is currently being evaluated in pancreatic cancer patients following surgical resection and adjuvant chemotherapy in an ongoing phase 1 clinical trial (NCT03122106). Neoantigen DNA vaccines incorporate prioritized neoantigens, and personalized MSLN epitopes will be administered intramuscularly using the TDS-IM system. The estimated completion date of this study is March 2022.

Despite the fact that there are few clinical trials of MSLN-targeted vaccines and the results of these trials have been disappointing, many preclinical studies are still ongoing. One study showed that a cell-based vaccine, Meso-VAX, in combination with the adeno-associated virus (AAV)-IL-12 increased the number of MSLN-specific T cells and the levels of anti-MSLN Abs and enhanced tumor clearance activity in mice [80]. The anti-tumor effects of the chimeric DNA vaccine CTGF/MSLN (containing an antigen-specific connective tissue growth factor linked to with MSLN) in combination with an anti-CD40 Ab and the TLR 3 ligand poly(I:C), which are essential adjuvants for DC maturation, the immuno-modulator EGCG and Meso-VAX in combination with (AAV)-IL-12 were proven [81]. Recently, a MSLN-derived epitope peptide restricted to HLA-A*2402 was shown to be effective in inducing peptide-specific CTLs. The MSLN-10-5 peptide-specific CTL clones showed specific cytotoxic activity against HLA-A*2402-positive MSLN-expressing pancreatic cancer cells, indicating that the peptide-based vaccine is a promising candidate for therapy [82].

## CAR-T therapy

### The development of MSLN-targeting CAR-T cells

Chimeric antigen receptor T (CAR-T) cells are designed to target cell surface antigens without MHC restriction. Therefore, the CAR-T cells could be broadly applicable in HLA-diverse allogeneic recipients. The CARs are recombinant receptors commonly consisting of an extracellular antigen recognition domain, which is generally derived from the single chain variable fragment (scFv) of antibodies, transmembrane domains that function as anchors in the cytoplasmic membrane, and an intracellular domain that transmits T cell activation signals. The first-generation CARs consisted of only one intracellular signaling domain, which was usually a CD3z chain, and this was sufficient to initiate T cell activation but produced only short-term proliferative activity and a low level of cytotoxicity. The second-generation CARs had greatly improved potency through the incorporation of another costimulatory molecule (CD28, 4-1BB, or OX40) [83–85]. Furthermore, our team and other groups demonstrated that the third-generation MSLN-targeting CARs containing two costimulatory domains (CD28, 4-1BB, TLR2, or DAP10) and a hinge domain were superior in terms of cell proliferation, cytotoxicity, persistence and tumor suppression efficacy [86–89]. The latest iteration, the fourth-generation CARs, can additionally secrete cytokines or other effector molecules, such as IL-12, IL-15, IL-7, CCL19, or αPD-1, to regulate the immune microenvironment [90–95].

Because MSLN is a highly specific antigen in several cancers, CAR-T therapy has been proven to be a promising strategy for the treatment of these cancers. TNBC is intractable due to the lack of an effective targeted therapy. The presence of MSLN in 67% of TNBCs provides a candidate target for CAR-T therapy of TNBC [23]. MSLN-directed CAR-T cells were demonstrated to induce cytotoxicity in MSLN-expressing pancreatic cancer cells in vivo depending on the MSLN expression level to delay tumor growth and eliminate lung metastases in vivo [96, 97]. Our team previously demonstrated that MSLN was also a promising target for treating lung cancer and gastric cancer [28, 87]. We proved that third-generation CAR-T could effectively delay tumor growth or even completely eradicate subcutaneous tumors, eliminate pulmonary and intraperitoneal metastases of gastric cancer cells in mice and prolong survival. Similarly, the effectiveness of this targeted strategy has also been proven in bile duct carcinoma [98] and ovarian cancer [99].

CAR-T cells are generally produced via lentivirus transduction. The CAR genes are cloned into lentiviral vectors and subsequently integrated into the host T cell genome, allowing for the stable and permanent expression of the CAR. This method has been widely adopted because it is simple and reliable. Another method used for the stable integration of the CAR gene into the T cell genome is the piggyBac transposon system. The piggyBac transposon system is an efficient nonviral method for the genomic engineering of mammalian cells, including pluripotent stem cells and human T lymphocytes, and its advantages include a large cargo capacity, nonrandom integration and the elimination of virus-associated issues [100]. MSLN-targeting CAR-T cells engineered by the piggyBac transposon system have been proven to be cytotoxic to pancreatic cancer cells [97] and bile duct carcinoma cells [98]. To avoid the risks associated with genomic integration, several studies have proposed that CAR-T cells targeting MSLN could be generated by RNA electroporation [99, 101]. The expression of the CAR was shown to be detectable 7 days after electroporation. Multiple injections of RNA-electroporated CAR T cells reduced tumor volumes in mice. However, the CAR is transiently expressed and will be completely eliminated over time as a result of the degradation of the CAR mRNA [99, 101].

CAR-T cells are generally administered by systemic delivery, such as intravenous injection. However, systemically delivered T cells need to pass through the barriers created by multiple tissues before infiltrating into tumors. Therefore, inefficient T cell infiltration and short persistence are common obstacles for solid tumor therapy by CAR-T. A recent preclinical study revealed that regional intrapleural administration of CAR T cells resulted in more robust proliferation and increased antitumor efficacy with a long persistence of 200 days in an orthotopic MPM model compared with that induced by systemically infused T cells [102]. Similarly, we found that the regional peritumoral delivery of CAR-T cells produced enhanced tumor clearance in a subcutaneous GC model [28]. The subcutaneous tumors in some mice in the peritumoral delivery group were completely eliminated, whereas a moderate effect was observed in the group treated with intravenously injected CAR-T cells. In addition, we found improved T cell infiltration in tumors in the peritumoral delivery group. Overall, regional delivery might enhance the therapeutic effects, but this requires verification in clinical trials. To enhance T cell infiltration, the MSLN-targeting CAR-T cells were also engineered to express CCR2b, a chemokine receptor that is minimally expressed on T cells, while the CCR2b ligand CCL2 is highly secreted by MPM [103]. The overexpression of CCR2b enhanced CAR-T cell cytotoxicity in tumor cells and chemotaxis in response to CCL2 in vitro. A 12.5-fold increase in T cell infiltration into tumors and significantly enhanced tumor clearance were observed in mice [103].

The tumor immune microenvironment is crucial in regulating T cell immunosurveillance. The upregulation of PD-L1 in tumor cells and the expression of inhibitory receptors, including PD1, CTLA-4, TIM3, LAG3, and 2B4, on T cells always reduces the infiltration of T cells into tumors and induces T cell exhaustion [95]. Recent preclinical studies showed that PD-1/PD-L1 blockade or CRISPR/Cas9-mediated PD-1 disruption could rescue MSLN-targeted CAR-T cell responses in vivo [104, 105]. Based on this, CAR-T cells engineered to express immune checkpoint antibodies (CTLA-4 and PD-1) or to knock out PD-1 are being evaluated in clinical trials [95] (NCT03030001, NCT03182803, NCT03615313, NCT03545815, and NCT03747965). In addition to being restricted by immune checkpoint molecules, the function of T cells is regulated by a variety of cytokines. The depletion of IL-10 with a blocking antibody or via the elevation of TNF-α and IL-2 levels by an oncolytic adenovirus enhanced and prolonged the functioning of MSLN-redirected CAR-T cells [106, 107].

MSLN-redirected CAR-T cells are also associated with the “on target, off tumor” issue. Despite the fact that no extensive or severe on-target toxicity against normal tissues has been observed, a great deal of effort has been made to avoid this problem. A promising strategy for this involves the achievement of accurate tumor recognition by combinatorial antigen-sensing circuits, while bispecific antibodies have proven more specific and potent [108]. Another potential approach is to physically separate the CD3ζ module from the costimulation module by using two distinct CARs specific for different antigens [109–111]. This structural design allows for comparable anticancer activity and persistence with the second-generation CAR-T cells only encounter both antigens. Another strategy is to engineer T cells with a synthetic Notch receptor that contains the core regulatory domain derived from the signaling receptor Notch [112]. An extracellular antigen recognition domain and a synthetic intracellular transcriptional domain were designed to replace the native Notch domain. Upon binding to the first antigen, the synthetic Notch receptor is cleaved and releases the intracellular transcriptional domain to activate the expression of the CAR, which recognizes the second antigen.

We have noted that the immunogenicity of murine-derived antibodies would limit their therapeutic effects in humans. Similarly, the use of a CAR of murine origin also limited the persistence of CAR-T cells in recipients. The development of a CAR with a human-derived scFv is needed to address this issue. A fully human MSLN-targeting CAR (P4) was constructed and shown to be enhanced in terms of cytokine secretion and cytotoxicity in vitro and anti-tumor activity in vivo [113]. P4 CAR-T cells were shown to be able to lyse MSLN-positive tumor cells in vitro and in vivo*,* even in the presence of soluble MSLN protein.

### Clinical trials of MSLN-targeting CAR-T cells

The majority of newly registered clinical trials targeting MSLN in the past 3 years are related to CAR-T therapy. CAR-T therapy has been a potent strategy for treating MSLN-expressing tumors [86, 114]. CAR design has been greatly optimized to enhance its performance [85]. The safety, effects and the maximum tolerated dose of MSLN-targeting CAR-T cell therapy are currently being evaluated in multiple phase 1/2 clinical trials (Table 2).

**Table 2:** Clinical trials for MSLN-targeted therapies based on CAR-T therapy

NCT Number	Title	Status	Interventions	Phases	Enrollment	Start Date	Locations
NCT03814447	The Fourth Generation CART-cell Therapy for Refractory-Relapsed Ovarian Cancer	Not yet recruiting	Drug: anti- MESO CAR-T cells; Drug: Fludarabine; Drug: Cyclophosphamide	Early Phase 1	10	2019-04-01	China
NCT03747965	Study of PD-1 Gene-knocked Out Mesothelin-directed CAR-T Cells With the Conditioning of PC in Mesothelin Positive Multiple Solid Tumors	Recruiting	Biological: Mesothelin-directed CAR-T cells	Phase 1	10	2018-11-01	China
NCT03608618	Intraperitoneal MCY-M11 (Mesothelin-targeting CAR) for Treatment of Advanced Ovarian Cancer and Peritoneal Mesothelioma	Recruiting	Biological: MCY-M11	Phase 1	15	2018-08-27	United States
NCT03615313	PD-1 Antibody Expressing mesoCAR-T Cells for Mesothelin Positive Advanced Solid Tumor	Recruiting	Biological: PD-1 antibody expressing mesoCAR-T cells	Phase 1/2	50	2018-08-06	China
NCT03638193	Study of Autologous T-cells in Patients With Metastatic Pancreatic Cancer	Recruiting	Biological: CART-meso cells	Not Applicable	10	2018-07-11	China
NCT03545815	Study of CRISPR-Cas9 Mediated PD-1 and TCR Gene-knocked Out Mesothelin-directed CAR-T Cells in Patients With Mesothelin Positive Multiple Solid Tumors.	Recruiting	Biological: anti-mesothelin CAR-T cells	Phase 1	10	2018-03-01	China
NCT03356808	Antigen-specific T Cells Against Lung Cancer	Recruiting	Biological: Lung cancer-specific T cells	Phase 1/2	20	2017-12-15	China
NCT03356795	Intervention of CAR-T Against Cervical Cancer	Recruiting	Biological: Cervical cancer-specific CAR-T cells	Phase 1/2	20	2017-11-15	China
NCT03497819	Autologous CARTmeso/19 Against Pancreatic Cancer	Active, not recruiting	Biological: CARTmeso CART19	Early Phase 1	10	2017-10-01	China
NCT03323944	CAR T Cell Immunotherapy for Pancreatic Cancer	Recruiting	Biological: huCART-meso cells	Phase 1	18	2017-09-15	United States
NCT03198052	HER2/Mesothelin/Lewis-Y/PSCA/MUC1/PD-L1/CD80/86-CAR-T Cells Immunotherapy Against Cancers	Recruiting	Biological: CAR-T cells targeting HER2, Mesothelin, PSCA, MUC1, Lewis-Y, or CD80/86	Phase 1	30	2017-07-01	China
NCT03267173	Evaluate the Safety and Efficacy of CAR-T in the Treatment of Pancreatic Cancer.	Recruiting	Drug: Chimeric antigen receptor T cell	Early Phase 1	10	2017-06-15	China
NCT03182803	CTLA-4 and PD-1 Antibodies Expressing Mesothelin-CAR-T Cells for Mesothelin Positive Advanced Solid Tumor	Recruiting	Biological: CTLA-4/PD-1 antibodies expressing mesoCAR-T	Phase 1/2	40	2017-06-07	China
NCT03054298	CAR T Cells in Mesothelin Expressing Cancers	Recruiting	Biological: huCART-meso cells	Phase 1	30	2017-03-01	United States
NCT03030001	PD-1 Antibody Expressing CAR T Cells for Mesothelin Positive Advanced Malignancies	Unknown status	Biological: PD-1 antibody expressing mesothelin specific CAR-T cells	Phase 1/2	40	2017-02-15	China
NCT02930993	Anti-mesothelin CAR T Cells for Patients With Recurrent or Metastatic Malignant Tumors	Recruiting	Biological: anti-mesothelin CAR T cells	Phase 1	20	2016-08-01	China
NCT02959151	A Study of Chimeric Antigen Receptor T Cells Combined With Interventional Therapy in Advanced Liver Malignancy	Unknown status	Drug: CAR-T cell	Phase 1/2	20	2016-07-01	China
NCT02792114	T-Cell Therapy for Advanced Breast Cancer	Recruiting	Drug: Cyclophosphamide; Biological: Mesothelin-targeted T cells; Drug: AP1903	Phase 1	36	2016-06-01	United States
NCT02706782	A Study of Mesothelin Redirected Autologous T Cells for Advanced Pancreatic Carcinoma	Unknown status	Drug: TAI-meso-CART	Phase 1	30	2016-03-01	China
NCT02580747	Treatment of Relapsed and/or Chemotherapy Refractory Advanced Malignancies by CART-meso	Unknown status	Biological: anti-meso-CAR vector transduced T cells	Phase 1	20	2015-10-01	China
NCT02414269	Malignant Pleural Disease Treated With Autologous T Cells Genetically Engineered to Target the Cancer-Cell Surface Antigen Mesothelin	Recruiting	Genetic: iCasp9M28z T cell infusions; Drug: cyclophosphamide	Phase 1	48	2015-05-01	United States
NCT02465983	Pilot Study of Autologous T-cells in Patients With Metastatic Pancreatic Cancer	Completed	Biological: CART-meso-19 T cells; Drug: Cyclophosphamide	Phase 1	4	2015-05-01	United States
NCT02388828	CART-meso Long-term Follow-up	Active, not recruiting	Biological: lentiviral-based CART meso therapy		10	2015-03-01	United States
NCT02159716	CART-meso in Mesothelin Expressing Cancers	Completed	Biological: CART-meso	Phase 1	19	2014-06-01	United States
NCT01897415	Autologous Redirected RNA Meso CAR T Cells for Pancreatic Cancer	Completed	Biological: Autologous T cells transfected with chimeric anti-mesothelin immunoreceptor SS1	Phase 1	16	2013-07-01	United States
NCT01583686	CAR T Cell Receptor Immunotherapy Targeting Mesothelin for Patients With Metastatic Cancer	Terminated	Drug: Fludarabine; Biological: Anti-mesothelin CAR transduced PBL; Drug: Cycolphosphamide; Drug: Aldesleukin	Phase 1/2	15	2012-05-04	United States
NCT01355965	Autologous Redirected RNA Meso-CIR T Cells	Completed	Biological: Autologous T cells	Phase 1	18	2011-03-01	United States

In a preclinical study, MSLN-targeting CAR-T cells generated by the transfection of mRNA showed robust antitumor activity and the transient expression of the CAR. mRNA-based CAR-T cells (SS1–4-1BB CAR) were proven to be well tolerated after multiple intravenous or intratumoral infusions (NCT01355965) [115, 116]. A confirmed partial response was observed in patients with MPM or PDA. The serum levels of inflammatory cytokines, including MIP-1β, granulocyte colony-stimulating factor (G-CSF), IL-6, and IL-17, were transiently elevated after each infusion of CAR-T cells [115]. CAR-T cells were detected in tumors with reduced CAR transcripts several days after administration. Notably, MSLN-targeting CAR-T cells were able to lyse primary tumor cells and elicit a systemic antitumor immune response by inducing epitope spreading [116].

In another recent phase 1 clinical trial, 6 patients with chemotherapy-refractory metastatic PDAC were intravenously administered autologous MSLN-targeting CAR-T cells 3 times weekly for 3 weeks [117]. Two patients had stable disease with PFS of 3.8 and 5.4 months. A decrease in MSLN expression by 69.2% in one patient was confirmed by biopsy. None of the 6 patients experienced cytokine release syndrome or neurological symptoms. Noteworthily, no evident on-target/off-tumor toxicity against normal tissues was observed in these patients [116, 117]. However, in addition to the short life span of the CAR, another issue that might limit its potency is the production of human anti-CAR antibodies [115–117]. An anaphylactic response reported in one patient was attributed to the high production of IgE antibodies specific to the CAR [115]. This suggests that a fully human anti-MSLN scFv is urgently needed for clinical use. Interestingly, a clinical trial that aims to impede the production of antibodies via the depletion of B cells by CD19-targeting CAR-T cells has been initiated (NCT03497819). This clinical trial is active but is not recruiting yet.

Regional delivery was proven to enhance T cell proliferation, persistence and function in mice. Because of this, regional delivery was applied to the clinical treatment of patients. CAR-T cells were administered intrapleurally, intratumorally, or by vascular interventional mediated injection (NCT02414269, NCT02706782, NCT02959151, NCT03267173, and NCT03198052). We still await the publication of the clinical outcomes to determine the importance of regional delivery in the clinic.

CAR-T therapy is always accompanied by cytokine release syndrome (CRS) and neurotoxicity due to the excessive immune activation of CAR-T or non-CAR-T cells, and the severity of this is associated with disease burden, the CAR-T cell dose, high-intensity lymphodepletion and preexisting endothelial activation [118]. To decrease the CAR-T-induced side effects, debulking chemotherapy is recommended to reduce tumor burden and the subsequent CAR-T dose, and tocilizumab could be used to prevent severe CRS in the clinic [118]. To enhance the safety of CAR-T therapy and controllably eliminate CAR-T cells when SAEs occur or tumors are eliminated, inducible suicide genes, including iCaspase-9, HSV-TK or EGFRΔ, could co-transduced with the CAR genes [25]. Exposure to a synthetic dimerizing drug would induce the dimerization of iCaspase-9 and lead to cell apoptosis. This inducible T-cell safety switch involving iCaspase-9 has been proven to eliminate over 90% of modified T cells within 30 min [119]. A MSLN-targeting CAR-T therapy trial involving the use of iCaspase-9 is currently recruiting (NCT03747965).

## Conclusions

The expression pattern of MSLN provides an exciting opportunity for its use in targeted therapy in various types of malignant tumors, including pancreatic cancer, ovarian cancer, lung cancer, TNBC and gastric cancer. To date, antibody-based drugs have been effective in inhibiting cancer progression and show acceptable “on target, off tumor” toxicity, while vaccines have showed moderate effects. The great improvements in CAR-T design allows them to be a promising therapeutic strategy to treat MSLN-expressing tumors. The immunogenicity of drugs and CAR-T cells, the low level of T cell infiltration into tumors and the high level of immunosuppression in the tumor microenvironment are obstacles that need to be overcome. The combined use with checkpoint inhibitors as well as additional strategies to reduce drug resistance and optimize delivery regimens might show promise in the future.

## Data Availability

Not applicable.

## Abbreviations

ADCC: Antibody-dependent cell-mediated cytotoxicity
ADCP: Antibody-dependent cell-mediated phagocytosis
cfu: Colony forming units
CRS: Cytokine release syndrome
DHAEs: Drug hypersensitivity adverse events
DLTs: Dose-limiting toxicities
G-CSF: Granulocyte colony-stimulating factor
GPI: Glycosylphosphatidylinositol
*Lm*: *Listeria monocytogenes*
MMAE: Monomethyl auristatin E
MPF: Megakaryocyte potentiating factor
MPM: Malignant pleural mesothelioma
MSLN: Mesothelin
MTD: Maximum tolerated dose
NSCLC: Non-small cell lung cancer
PD: Progressive disease
PDA: Pancreatic ductal carcinoma
PE: *Pseudomonas* exotoxin
PK: Pharmacokinetics
PLD: Pegylated liposomal doxorubicin
PR: Partial responses
RIT: Recombination immunotoxin
scFv: Single chain variable fragment
SD: Stable disease
SMRP: Soluble MSLN-related protein
TNBC: Triple negative breast cancer

## References

[1] Yamaguchi N, Hattori K, Oh-eda M, Kojima T, Imai N, Ochi N (1994). A novel cytokine exhibiting megakaryocyte potentiating activity from a human pancreatic tumor cell line HPC-Y5. J Biol Chem.

[2] Chang K, Pastan I, Willingham MC (1992). Isolation and characterization of a monoclonal antibody, K1, reactive with ovarian cancers and normal mesothelium. Int J Cancer.

[3] Ordonez NG (2003). Application of mesothelin immunostaining in tumor diagnosis. Am J Surg Pathol.

[4] Bera TK, Pastan I (2000). Mesothelin is not required for normal mouse development or reproduction. Mol Cell Biol.

[5] Servais EL, Colovos C, Rodriguez L, Bograd AJ, Nitadori J, Sima C, Rusch VW, Sadelain M, Adusumilli PS (2012). Mesothelin overexpression promotes mesothelioma cell invasion and MMP-9 secretion in an orthotopic mouse model and in epithelioid pleural mesothelioma patients. Clin Cancer Res.

[6] Rump A, Morikawa Y, Tanaka M, Minami S, Umesaki N, Takeuchi M, Miyajima A (2004). Binding of ovarian cancer antigen CA125/MUC16 to mesothelin mediates cell adhesion. J Biol Chem.

[7] Gubbels JA, Belisle J, Onda M, Rancourt C, Migneault M, Ho M, Bera TK, Connor J, Sathyanarayana BK, Lee B, Pastan I, Patankar MS (2006). Mesothelin-MUC16 binding is a high affinity, N-glycan dependent interaction that facilitates peritoneal metastasis of ovarian tumors. Mol Cancer.

[8] Coelho R, Marcos-Silva L, Ricardo S, Ponte F, Costa A, Lopes JM, David L (2018). Peritoneal dissemination of ovarian cancer: role of MUC16-mesothelin interaction and implications for treatment. Expert Rev Anticancer Ther.

[9] Chen SH, Hung WC, Wang P, Paul C, Konstantopoulos K (2013). Mesothelin binding to CA125/MUC16 promotes pancreatic cancer cell motility and invasion via MMP-7 activation. Sci Rep.

[10] Bharadwaj U, Marin-Muller C, Li M, Chen C, Yao Q (2011). Mesothelin confers pancreatic cancer cell resistance to TNF-alpha-induced apoptosis through Akt/PI3K/NF-kappaB activation and IL-6/Mcl-1 overexpression. Mol Cancer.

[11] Kachala SS, Bograd AJ, Villena-Vargas J, Suzuki K, Servais EL, Kadota K, Chou J, Sima CS, Vertes E, Rusch VW, Travis WD, Sadelain M, Adusumilli PS (2014). Mesothelin overexpression is a marker of tumor aggressiveness and is associated with reduced recurrence-free and overall survival in early-stage lung adenocarcinoma. Clin Cancer Res.

[12] Tozbikian G, Brogi E, Kadota K, Catalano J, Akram M, Patil S, Ho AY, Reis-Filho JS, Weigelt B, Norton L, Adusumilli PS, Wen HY (2014). Mesothelin expression in triple negative breast carcinomas correlates significantly with basal-like phenotype, distant metastases and decreased survival. PLoS One.

[13] Sathyanarayana BK, Hahn Y, Patankar MS, Pastan I, Lee B (2009). Mesothelin, Stereocilin, and Otoancorin are predicted to have superhelical structures with ARM-type repeats. BMC Struct Biol.

[14] Ma J, Tang WK, Esser L, Pastan I, Xia D (2012). Characterization of crystals of an antibody-recognition fragment of the cancer differentiation antigen mesothelin in complex with the therapeutic antibody MORAb-009. Acta Crystallogr Sect F Struct Biol Cryst Commun.

[15] Chang K, Pastan I (1996). Molecular cloning of mesothelin, a differentiation antigen present on mesothelium, mesotheliomas, and ovarian cancers. Proc Natl Acad Sci U S A.

[16] Chang K, Pai LH, Pass H, Pogrebniak HW, Tsao MS, Pastan I, Willingham MC (1992). Monoclonal antibody K1 reacts with epithelial mesothelioma but not with lung adenocarcinoma. Am J Surg Pathol.

[17] Ordonez NG (2003). Value of mesothelin immunostaining in the diagnosis of mesothelioma. Mod Pathol.

[18] Miettinen M, Sarlomo-Rikala M (2003). Expression of calretinin, thrombomodulin, keratin 5, and mesothelin in lung carcinomas of different types: an immunohistochemical analysis of 596 tumors in comparison with epithelioid mesotheliomas of the pleura. Am J Surg Pathol.

[19] Argani P, Iacobuzio-Donahue C, Ryu B, Rosty C, Goggins M, Wilentz RE, Murugesan SR, Leach SD, Jaffee E, Yeo CJ, Cameron JL, Kern SE, Hruban RH (2001). Mesothelin is overexpressed in the vast majority of ductal adenocarcinomas of the pancreas: identification of a new pancreatic cancer marker by serial analysis of gene expression (SAGE). Clin Cancer Res.

[20] Hassan R, Laszik ZG, Lerner M, Raffeld M, Postier R, Brackett D (2005). Mesothelin is overexpressed in pancreaticobiliary adenocarcinomas but not in normal pancreas and chronic pancreatitis. Am J Clin Pathol.

[21] O'Hara MH, Stashwick C, Plesa G, Tanyi JL (2017). Overcoming barriers of car T-cell therapy in patients with mesothelin-expressing cancers. Immunotherapy.

[22] Chang K, Pastan I, Willingham MC (1992). Frequent expression of the tumor antigen CAK1 in squamous-cell carcinomas. Int J Cancer.

[23] Tchou J, Wang LC, Selven B, Zhang H, Conejo-Garcia J, Borghaei H, Kalos M, Vondeheide RH, Albelda SM, June CH, Zhang PJ (2012). Mesothelin, a novel immunotherapy target for triple negative breast cancer. Breast Cancer Res Treat.

[24] Rizk NP, Servais EL, Tang LH, Sima CS, Gerdes H, Fleisher M, Rusch VW, Adusumilli PS (2012). Tissue and serum mesothelin are potential markers of neoplastic progression in Barrett's associated esophageal adenocarcinoma. Cancer Epidemiol Biomark Prev.

[25] Morello A, Sadelain M, Adusumilli PS (2016). Mesothelin-targeted CARs: driving T cells to solid tumors. Cancer Discov.

[26] Pastan I, Hassan R (2014). Discovery of mesothelin and exploiting it as a target for immunotherapy. Cancer Res.

[27] Han SH, Joo M, Kim H, Chang S (2017). Mesothelin expression in gastric adenocarcinoma and its relation to clinical outcomes. J Pathol Transl Med.

[28] Lv J, Zhao R, Wu D, Zheng D, Wu Z, Shi J, Wei X, Wu Q, Long Y, Lin S, Wang S, Wang Z, Li Y, Chen Y, He Q, Chen S, Yao H, Liu Z, Tang Z, Yao Y, Pei D, Liu P, Zhang X, Zhang Z, Cui S, Chen R, Li P (2019). Mesothelin is a target of chimeric antigen receptor T cells for treating gastric cancer. J Hematol Oncol.

[29] Cheng WF, Huang CY, Chang MC, Hu YH, Chiang YC, Chen YL, Hsieh CY, Chen CA (2009). High mesothelin correlates with chemoresistance and poor survival in epithelial ovarian carcinoma. Br J Cancer.

[30] Kawamata F, Kamachi H, Einama T, Homma S, Tahara M, Miyazaki M, Tanaka S, Kamiyama T, Nishihara H, Taketomi A, Todo S (2012). Intracellular localization of mesothelin predicts patient prognosis of extrahepatic bile duct cancer. Int J Oncol.

[31] Nomura R, Fujii H, Abe M, Sugo H, Ishizaki Y, Kawasaki S, Hino O (2013). Mesothelin expression is a prognostic factor in cholangiocellular carcinoma. Int Surg.

[32] Thomas A, Chen Y, Steinberg SM, Luo J, Pack S, Raffeld M, Abdullaev Z, Alewine C, Rajan A, Giaccone G, Pastan I, Miettinen M, Hassan R (2015). High mesothelin expression in advanced lung adenocarcinoma is associated with KRAS mutations and a poor prognosis. Oncotarget.

[33] Li YR, Xian RR, Ziober A, Conejo-Garcia J, Perales-Puchalt A, June CH, Zhang PJ, Tchou J (2014). Mesothelin expression is associated with poor outcomes in breast cancer. Breast Cancer Res Treat.

[34] Winter JM, Tang LH, Klimstra DS, Brennan MF, Brody JR, Rocha FG, Jia X, Qin LX, D'Angelica MI, DeMatteo RP, Fong Y, Jarnagin WR, O'Reilly EM, Allen PJ (2012). A novel survival-based tissue microarray of pancreatic cancer validates MUC1 and mesothelin as biomarkers. PLoS One.

[35] Shimizu A, Hirono S, Tani M, Kawai M, Okada K, Miyazawa M, Kitahata Y, Nakamura Y, Noda T, Yokoyama S, Yamaue H (2012). Coexpression of MUC16 and mesothelin is related to the invasion process in pancreatic ductal adenocarcinoma. Cancer Sci.

[36] Hassan R, Thomas A, Alewine C, Le DT, Jaffee EM, Pastan I (2016). Mesothelin immunotherapy for Cancer: ready for prime time?. J Clin Oncol.

[37] Cristaudo A, Bonotti A, Guglielmi G, Fallahi P, Foddis R (2018). Serum mesothelin and other biomarkers: what have we learned in the last decade?. J Thorac Dis.

[38] Sapede C, Gauvrit A, Barbieux I, Padieu M, Cellerin L, Sagan C, Scherpereel A, Dabouis G, Gregoire M (2008). Aberrant splicing and protease involvement in mesothelin release from epithelioid mesothelioma cells. Cancer Sci.

[39] Robinson BW, Creaney J, Lake R, Nowak A, Musk AW, de Klerk N, Winzell P, Hellstrom KE, Hellstrom I (2003). Mesothelin-family proteins and diagnosis of mesothelioma. Lancet.

[40] Bast RC (2003). Status of tumor markers in ovarian cancer screening. J Clin Oncol.

[41] Hassan R, Wu C, Brechbiel MW, Margulies I, Kreitman RJ, Pastan I (1999). 111Indium-labeled monoclonal antibody K1: biodistribution study in nude mice bearing a human carcinoma xenograft expressing mesothelin. Int J Cancer.

[42] Hassan R, Viner JL, Wang QC, Margulies I, Kreitman RJ, Pastan I (2000). Anti-tumor activity of K1-LysPE38QQR, an immunotoxin targeting mesothelin, a cell-surface antigen overexpressed in ovarian cancer and malignant mesothelioma. J Immunother.

[43] Chowdhury PS, Viner JL, Beers R, Pastan I (1998). Isolation of a high-affinity stable single-chain Fv specific for mesothelin from DNA-immunized mice by phage display and construction of a recombinant immunotoxin with anti-tumor activity. Proc Natl Acad Sci U S A.

[44] Chowdhury PS, Pastan I (1999). Improving antibody affinity by mimicking somatic hypermutation in vitro. Nat Biotechnol.

[45] Hilliard Tyvette (2018). The Impact of Mesothelin in the Ovarian Cancer Tumor Microenvironment. Cancers.

[46] Hassan R, Sharon E, Thomas A, Zhang J, Ling A, Miettinen M, Kreitman RJ, Steinberg SM, Hollevoet K, Pastan I (2014). Phase 1 study of the antimesothelin immunotoxin SS1P in combination with pemetrexed and cisplatin for front-line therapy of pleural mesothelioma and correlation of tumor response with serum mesothelin, megakaryocyte potentiating factor, and cancer antigen 125. Cancer.

[47] Hassan R, Miller AC, Sharon E, Thomas A, Reynolds JC, Ling A, Kreitman RJ, Miettinen MM, Steinberg SM, Fowler DH, Pastan I (2013). Major cancer regressions in mesothelioma after treatment with an anti-mesothelin immunotoxin and immune suppression. Sci Transl Med.

[48] Hassan R, Bullock S, Premkumar A, Kreitman RJ, Kindler H, Willingham MC, Pastan I (2007). Phase I study of SS1P, a recombinant anti-mesothelin immunotoxin given as a bolus I.V. infusion to patients with mesothelin-expressing mesothelioma, ovarian, and pancreatic cancers. Clin Cancer Res.

[49] Kreitman RJ, Hassan R, Fitzgerald DJ, Pastan I (2009). Phase I trial of continuous infusion anti-mesothelin recombinant immunotoxin SS1P. Clin Cancer Res.

[50] Gupta A, Hussein Z, Hassan R, Wustner J, Maltzman JD, Wallin BA (2016). Population pharmacokinetics and exposure-response relationship of amatuximab, an anti-mesothelin monoclonal antibody, in patients with malignant pleural mesothelioma and its application in dose selection. Cancer Chemother Pharmacol.

[51] Fujisaka Y, Kurata T, Tanaka K, Kudo T, Okamoto K, Tsurutani J, Kaneda H, Okamoto I, Namiki M, Kitamura C, Nakagawa K (2015). Phase I study of amatuximab, a novel monoclonal antibody to mesothelin, in Japanese patients with advanced solid tumors. Investig New Drugs.

[52] Hassan R, Cohen SJ, Phillips M, Pastan I, Sharon E, Kelly RJ, Schweizer C, Weil S, Laheru D (2010). Phase I clinical trial of the chimeric anti-mesothelin monoclonal antibody MORAb-009 in patients with mesothelin-expressing cancers. Clin Cancer Res.

[53] Hassan R, Schweizer C, Lu KF, Schuler B, Remaley AT, Weil SC, Pastan I (2010). Inhibition of mesothelin-CA-125 interaction in patients with mesothelioma by the anti-mesothelin monoclonal antibody MORAb-009: implications for cancer therapy. Lung Cancer.

[54] Hassan R, Kindler HL, Jahan T, Bazhenova L, Reck M, Thomas A, Pastan I, Parno J, O'Shannessy DJ, Fatato P, Maltzman JD, Wallin BA (2014). Phase II clinical trial of amatuximab, a chimeric antimesothelin antibody with pemetrexed and cisplatin in advanced unresectable pleural mesothelioma. Clin Cancer Res.

[55] Quanz M, Hagemann UB, Zitzmann-Kolbe S, Stelte-Ludwig B, Golfier S, Elbi C, Mumberg D, Ziegelbauer K, Schatz CA (2018). Anetumab ravtansine inhibits tumor growth and shows additive effect in combination with targeted agents and chemotherapy in mesothelin-expressing human ovarian cancer models. Oncotarget.

[56] Golfier S, Kopitz C, Kahnert A, Heisler I, Schatz CA, Stelte-Ludwig B, Mayer-Bartschmid A, Unterschemmann K, Bruder S, Linden L, Harrenga A, Hauff P, Scholle FD, Muller-Tiemann B, Kreft B, Ziegelbauer K (2014). Anetumab ravtansine: a novel mesothelin-targeting antibody-drug conjugate cures tumors with heterogeneous target expression favored by bystander effect. Mol Cancer Ther.

[57] Baldo P, Cecco S (2017). Amatuximab and novel agents targeting mesothelin for solid tumors. Onco Targets Ther.

[58] Le DT, Wang-Gillam A, Picozzi V, Greten TF, Crocenzi T, Springett G, Morse M, Zeh H, Cohen D, Fine RL, Onners B, Uram JN, Laheru DA, Lutz ER, Solt S, Murphy AL, Skoble J, Lemmens E, Grous J, Dubensky T, Brockstedt DG, Jaffee EM (2015). Safety and survival with GVAX pancreas prime and Listeria monocytogenes-expressing mesothelin (CRS-207) boost vaccines for metastatic pancreatic cancer. J Clin Oncol.

[59] Le DT, Brockstedt DG, Nir-Paz R, Hampl J, Mathur S, Nemunaitis J, Sterman DH, Hassan R, Lutz E, Moyer B, Giedlin M, Louis JL, Sugar EA, Pons A, Cox AL, Levine J, Murphy AL, Illei P, Dubensky TW, Eiden JE, Jaffee EM, Laheru DA (2012). A live-attenuated Listeria vaccine (ANZ-100) and a live-attenuated Listeria vaccine expressing mesothelin (CRS-207) for advanced cancers: phase I studies of safety and immune induction. Clin Cancer Res.

[60] Lindenberg L, Thomas A, Adler S, Mena E, Kurdziel K, Maltzman J, Wallin B, Hoffman K, Pastan I, Paik CH, Choyke P, Hassan R (2015). Safety and biodistribution of 111In-amatuximab in patients with mesothelin expressing cancers using single photon emission computed tomography-computed tomography (SPECT-CT) imaging. Oncotarget.

[61] Scales SJ, Gupta N, Pacheco G, Firestein R, French DM, Koeppen H, Rangell L, Barry-Hamilton V, Luis E, Chuh J, Zhang Y, Ingle GS, Fourie-O'Donohue A, Kozak KR, Ross S, Dennis MS, Spencer SD (2014). An antimesothelin-monomethyl auristatin e conjugate with potent antitumor activity in ovarian, pancreatic, and mesothelioma models. Mol Cancer Ther.

[62] Weekes CD, Lamberts LE, Borad MJ, Voortman J, McWilliams RR, Diamond JR, de Vries EG, Verheul HM, Lieu CH, Kim GP, Wang Y, Scales SJ, Samineni D, Brunstein F, Choi Y, Maslyar DJ, Colon-Otero G (2016). Phase I study of DMOT4039A, an antibody-drug conjugate targeting Mesothelin, in patients with Unresectable pancreatic or platinum-resistant ovarian Cancer. Mol Cancer Ther.

[63] Hollevoet K, Mason-Osann E, Liu XF, Imhof-Jung S, Niederfellner G, Pastan I (2014). In vitro and in vivo activity of the low-immunogenic antimesothelin immunotoxin RG7787 in pancreatic cancer. Mol Cancer Ther.

[64] El-Behaedi Salma, Landsman Rebekah, Rudloff Michael, Kolyvas Emily, Albalawy Rakan, Zhang Xianyu, Bera Tapan, Collins Keith, Kozlov Serguei, Alewine Christine (2018). Protein Synthesis Inhibition Activity of Mesothelin Targeting Immunotoxin LMB-100 Decreases Concentrations of Oncogenic Signaling Molecules and Secreted Growth Factors. Toxins.

[65] Ali-Rahmani F, FitzGerald DJ, Martin S, Patel P, Prunotto M, Ormanoglu P, Thomas C, Pastan I (2016). Anticancer effects of Mesothelin-targeted immunotoxin therapy are regulated by tyrosine kinase DDR1. Cancer Res.

[66] Alewine C, Xiang L, Yamori T, Niederfellner G, Bosslet K, Pastan I (2014). Efficacy of RG7787, a next-generation mesothelin-targeted immunotoxin, against triple-negative breast and gastric cancers. Mol Cancer Ther.

[67] Liu XF, Xiang L, Zhou Q, Carralot JP, Prunotto M, Niederfellner G, Pastan I (2016). Actinomycin D enhances killing of cancer cells by immunotoxin RG7787 through activation of the extrinsic pathway of apoptosis. Proc Natl Acad Sci U S A.

[68] Zhang J, Khanna S, Jiang Q, Alewine C, Miettinen M, Pastan I, Hassan R (2017). Efficacy of anti-mesothelin immunotoxin RG7787 plus nab-paclitaxel against mesothelioma patient-derived xenografts and Mesothelin as a biomarker of tumor response. Clin Cancer Res.

[69] Kolyvas E, Rudloff M, Poruchynsky M, Landsman R, Hollevoet K, Venzon D, Alewine C (2017). Mesothelin-targeted immunotoxin RG7787 has synergistic anti-tumor activity when combined with taxanes. Oncotarget.

[70] Liu XF, Zhou Q, Hassan R, Pastan I (2017). Panbinostat decreases cFLIP and enhances killing of cancer cells by immunotoxin LMB-100 by stimulating the extrinsic apoptotic pathway. Oncotarget.

[71] Wickstroem K, Hagemann UB, Cruciani V, Wengner AM, Kristian A, Ellingsen C, Siemeister G, Bjerke R, Karlsson J, Ryan OB, Linden L, Mumberg D, Ziegelbauer K, Cuthbertson AS. Synergistic effect of a Mesothelin targeted Thorium-227 conjugate in combination with DNA damage response inhibitors in ovarian Cancer xenograft models. J Nucl Med. 2019. https://www.ncbi.nlm.nih.gov/pubmed/30850485. http://jnm.snmjournals.org/citmgr?gca=jnumed%3Bjnumed.118.223701v1. 10.2967/jnumed.118.223701 PMC6735281 30850485

[72] Richard Austin WA, Patrick A. Baeuerle, Adrie Jones, Susan D. Jones, Che-Leung Law, Kathryn Kwant, Bryan Lemon, Anna Muchnik, Kenneth Sexton, Laurie Tatalick, Holger Wesche, Timothy Yu, Harpoon Therapeutics.: HPN536, a T cell-engaging, Mesothelin/CD3-specific TriTAC for the treatment of solid tumors. 2018. https://www.harpoontx.com/file.cfm/43/docs/AACR_2018_Poster_HPN536.pdf.

[73] Kaplan Gilad, Lee Fred, Onda Masanori, Kolyvas Emily, Bhardwaj Gaurav, Baker David, Pastan Ira (2016). Protection of the Furin Cleavage Site in Low-Toxicity Immunotoxins Based on Pseudomonas Exotoxin A. Toxins.

[74] Wei J, Bera TK, Liu XF, Zhou Q, Onda M, Ho M, Tai CH, Pastan I (2018). Recombinant immunotoxins with albumin-binding domains have long half-lives and high antitumor activity. Proc Natl Acad Sci U S A.

[75] Mazor R, Onda M, Park D, Addissie S, Xiang L, Zhang J, Hassan R, Pastan I (2016). Dual B- and T-cell de-immunization of recombinant immunotoxin targeting mesothelin with high cytotoxic activity. Oncotarget.

[76] Mazor R, Zhang J, Xiang L, Addissie S, Awuah P, Beers R, Hassan R, Pastan I (2015). Recombinant immunotoxin with T-cell epitope mutations that greatly reduce immunogenicity for treatment of Mesothelin-expressing tumors. Mol Cancer Ther.

[77] Feng Y, Xiao X, Zhu Z, Streaker E, Ho M, Pastan I, Dimitrov DS (2009). A novel human monoclonal antibody that binds with high affinity to mesothelin-expressing cells and kills them by antibody-dependent cell-mediated cytotoxicity. Mol Cancer Ther.

[78] Ho M, Feng M, Fisher RJ, Rader C, Pastan I (2011). A novel high-affinity human monoclonal antibody to mesothelin. Int J Cancer.

[79] Butterfield LH (2015). Cancer vaccines. Bmj.

[80] Chang MC, Chen YL, Chiang YC, Chen TC, Tang YC, Chen CA, Sun WZ, Cheng WF (2016). Mesothelin-specific cell-based vaccine generates antigen-specific immunity and potent antitumor effects by combining with IL-12 immunomodulator. Gene Ther.

[81] Chen YL, Chang MC, Chiang YC, Lin HW, Sun NY, Chen CA, Sun WZ, Cheng WF (2018). Immuno-modulators enhance antigen-specific immunity and anti-tumor effects of mesothelin-specific chimeric DNA vaccine through promoting DC maturation. Cancer Lett.

[82] Tsukagoshi M, Wada S, Hirono S, Yoshida S, Yada E, Sasada T, Shirabe K, Kuwano H, Yamaue H (2018). Identification of a novel HLA-A24-restricted cytotoxic T lymphocyte epitope peptide derived from mesothelin in pancreatic cancer. Oncotarget.

[83] Sadelain M, Brentjens R, Riviere I (2013). The basic principles of chimeric antigen receptor design. Cancer Discov.

[84] Zhang C, Liu J, Zhong JF, Zhang X (2017). Engineering CAR-T cells. Biomark Res.

[85] Wang Z, Wu Z, Liu Y, Han W (2017). New development in CAR-T cell therapy. J Hematol Oncol.

[86] Yu S, Li A, Liu Q, Li T, Yuan X, Han X, Wu K (2017). Chimeric antigen receptor T cells: a novel therapy for solid tumors. J Hematol Oncol.

[87] Lai Y, Weng J, Wei X, Qin L, Lai P, Zhao R, Jiang Z, Li B, Lin S, Wang S, Wu Q, Tang Z, Liu P, Pei D, Yao Y, Du X, Li P (2018). Toll-like receptor 2 costimulation potentiates the antitumor efficacy of CAR T cells. Leukemia.

[88] Zhao R, Cheng L, Jiang Z, Wei X, Li B, Wu Q, Wang S, Lin S, Long Y, Zhang X, Wu Y, Du X, Pei D, Liu P, Li Y, Cui S, Yao Y, Li P (2019). DNAX-activating protein 10 co-stimulation enhances the anti-tumor efficacy of chimeric antigen receptor T cells. Oncoimmunology.

[89] Qin L, Lai Y, Zhao R, Wei X, Weng J, Lai P, Li B, Lin S, Wang S, Wu Q, Liang Q, Li Y, Zhang X, Wu Y, Liu P, Yao Y, Pei D, Du X, Li P (2017). Incorporation of a hinge domain improves the expansion of chimeric antigen receptor T cells. J Hematol Oncol.

[90] Adachi K, Kano Y, Nagai T, Okuyama N, Sakoda Y, Tamada K (2018). IL-7 and CCL19 expression in CAR-T cells improves immune cell infiltration and CAR-T cell survival in the tumor. Nat Biotechnol.

[91] Li S, Siriwon N, Zhang X, Yang S, Jin T, He F, Kim YJ, Mac J, Lu Z, Wang S, Han X, Wang P (2017). Enhanced Cancer immunotherapy by chimeric antigen receptor-modified T cells engineered to secrete checkpoint inhibitors. Clin Cancer Res.

[92] Markley JC, Sadelain M (2010). IL-7 and IL-21 are superior to IL-2 and IL-15 in promoting human T cell-mediated rejection of systemic lymphoma in immunodeficient mice. Blood.

[93] Pegram HJ, Lee JC, Hayman EG, Imperato GH, Tedder TF, Sadelain M, Brentjens RJ (2012). Tumor-targeted T cells modified to secrete IL-12 eradicate systemic tumors without need for prior conditioning. Blood.

[94] Qin L, Zhao R, Li P (2017). Incorporation of functional elements enhances the antitumor capacity of CAR T cells. Exp Hematol Oncol.

[95] Li J, Li W, Huang K, Zhang Y, Kupfer G, Zhao Q (2018). Chimeric antigen receptor T cell (CAR-T) immunotherapy for solid tumors: lessons learned and strategies for moving forward. J Hematol Oncol.

[96] Sun Q, Zhou S, Zhao J, Deng C, Teng R, Zhao Y, Chen J, Dong J, Yin M, Bai Y, Deng H, Wen J (2018). Engineered T lymphocytes eliminate lung metastases in models of pancreatic cancer. Oncotarget.

[97] He J, Zhang Z, Lv S, Liu X, Cui L, Jiang D, Zhang Q, Li L, Qin W, Jin H, Qian Q (2018). Engineered CAR T cells targeting mesothelin by piggyBac transposon system for the treatment of pancreatic cancer. Cell Immunol.

[98] Xu JY, Ye ZL, Jiang DQ, He JC, Ding YM, Li LF, Lv SQ, Wang Y, Jin HJ, Qian QJ (2017). Mesothelin-targeting chimeric antigen receptor-modified T cells by piggyBac transposon system suppress the growth of bile duct carcinoma. Tumour Biol.

[99] Hung CF, Xu X, Li L, Ma Y, Jin Q, Viley A, Allen C, Natarajan P, Shivakumar R, Peshwa MV, Emens LA (2018). Development of anti-human Mesothelin-targeted chimeric antigen receptor messenger RNA-transfected peripheral blood lymphocytes for ovarian Cancer therapy. Hum Gene Ther.

[100] Woodard LE, Wilson MH (2015). piggyBac-ing models and new therapeutic strategies. Trends Biotechnol.

[101] Zhao Y, Moon E, Carpenito C, Paulos CM, Liu X, Brennan AL, Chew A, Carroll RG, Scholler J, Levine BL, Albelda SM, June CH (2010). Multiple injections of electroporated autologous T cells expressing a chimeric antigen receptor mediate regression of human disseminated tumor. Cancer Res.

[102] Adusumilli PS, Cherkassky L, Villena-Vargas J, Colovos C, Servais E, Plotkin J, Jones DR, Sadelain M (2014). Regional delivery of mesothelin-targeted CAR T cell therapy generates potent and long-lasting CD4-dependent tumor immunity. Sci Transl Med.

[103] Moon EK, Carpenito C, Sun J, Wang LC, Kapoor V, Predina J, Powell DJ, Riley JL, June CH, Albelda SM (2011). Expression of a functional CCR2 receptor enhances tumor localization and tumor eradication by retargeted human T cells expressing a mesothelin-specific chimeric antibody receptor. Clin Cancer Res.

[104] Cherkassky L, Morello A, Villena-Vargas J, Feng Y, Dimitrov DS, Jones DR, Sadelain M, Adusumilli PS (2016). Human CAR T cells with cell-intrinsic PD-1 checkpoint blockade resist tumor-mediated inhibition. J Clin Invest.

[105] Hu W, Zi Z, Jin Y, Li G, Shao K, Cai Q, Ma X, Wei F (2019). CRISPR/Cas9-mediated PD-1 disruption enhances human mesothelin-targeted CAR T cell effector functions. Cancer Immunol Immunother.

[106] Batchu RB, Gruzdyn OV, Mahmud EM, Chukr F, Dachepalli R, Manmari SK, Mostafa G, Weaver DW, Gruber SA (2018). Inhibition of Interleukin-10 in the tumor microenvironment can restore mesothelin chimeric antigen receptor T cell activity in pancreatic cancer in vitro. Surgery.

[107] Watanabe K, Luo Y, Da T, Guedan S, Ruella M, Scholler J, Keith B, Young RM, Engels B, Sorsa S, Siurala M, Havunen R, Tahtinen S, Hemminki A, June CH. Pancreatic cancer therapy with combined mesothelin-redirected chimeric antigen receptor T cells and cytokine-armed oncolytic adenoviruses. JCI Insight. 2018;3(7):e99573. 10.1172/jci.insight.99573 PMC5928866 29618658

[108] Zhang X, Yang Y, Fan D, Xiong D (2017). The development of bispecific antibodies and their applications in tumor immune escape. Exp Hematol Oncol.

[109] Lanitis E, Poussin M, Klattenhoff AW, Song D, Sandaltzopoulos R, June CH, Powell DJ (2013). Chimeric antigen receptor T cells with dissociated signaling domains exhibit focused antitumor activity with reduced potential for toxicity in vivo. Cancer Immunol Res.

[110] Zhang E, Yang P, Gu J, Wu H, Chi X, Liu C, Wang Y, Xue J, Qi W, Sun Q, Zhang S, Hu J, Xu H (2018). Recombination of a dual-CAR-modified T lymphocyte to accurately eliminate pancreatic malignancy. J Hematol Oncol.

[111] Zhang E, Gu J, Xue J, Lin C, Liu C, Li M, Hao J, Setrerrahmane S, Chi X, Qi W, Hu J, Xu H (2018). Accurate control of dual-receptor-engineered T cell activity through a bifunctional anti-angiogenic peptide. J Hematol Oncol.

[112] Roybal KT, Rupp LJ, Morsut L, Walker WJ, McNally KA, Park JS, Lim WA (2016). Precision tumor recognition by T cells with combinatorial antigen-sensing circuits. Cell.

[113] Lanitis E, Poussin M, Hagemann IS, Coukos G, Sandaltzopoulos R, Scholler N, Powell DJ (2012). Redirected antitumor activity of primary human lymphocytes transduced with a fully human anti-mesothelin chimeric receptor. Mol Ther.

[114] Liu B, Song Y, Liu D (2017). Clinical trials of CAR-T cells in China. J Hematol Oncol.

[115] Maus MV, Haas AR, Beatty GL, Albelda SM, Levine BL, Liu X, Zhao Y, Kalos M, June CH (2013). T cells expressing chimeric antigen receptors can cause anaphylaxis in humans. Cancer Immunol Res.

[116] Beatty GL, Haas AR, Maus MV, Torigian DA, Soulen MC, Plesa G, Chew A, Zhao Y, Levine BL, Albelda SM, Kalos M, June CH (2014). Mesothelin-specific chimeric antigen receptor mRNA-engineered T cells induce anti-tumor activity in solid malignancies. Cancer Immunol Res.

[117] Beatty GL, O'Hara MH, Lacey SF, Torigian DA, Nazimuddin F, Chen F, Kulikovskaya IM, Soulen MC, McGarvey M, Nelson AM, Gladney WL, Levine BL, Melenhorst JJ, Plesa G, June CH (2018). Activity of Mesothelin-specific chimeric antigen receptor T cells against pancreatic carcinoma metastases in a phase 1 trial. Gastroenterology.

[118] Wang Z, Han W (2018). Biomarkers of cytokine release syndrome and neurotoxicity related to CAR-T cell therapy. Biomark Res.

[119] Di Stasi A, Tey SK, Dotti G, Fujita Y, Kennedy-Nasser A, Martinez C, Straathof K, Liu E, Durett AG, Grilley B, Liu H, Cruz CR, Savoldo B, Gee AP, Schindler J, Krance RA, Heslop HE, Spencer DM, Rooney CM, Brenner MK (2011). Inducible apoptosis as a safety switch for adoptive cell therapy. N Engl J Med.

